# Prevalence and risk factors of intestinal schistosomiasis on students at Bochesa Elementary School, around the wetlands of Lake Ziway, Ethiopia

**DOI:** 10.1016/j.ijregi.2025.100591

**Published:** 2025-02-01

**Authors:** Ayalew Sisay

**Affiliations:** Department of Biology, College of Natural and Computational Sciences, Debre Markos University, Debre Markos, Ethiopia

**Keywords:** Intestinal schistosomiasis, Students, Kato-Katz, Wetland, Ziway

## Abstract

**Objectives:**

This study aimed to assess the prevalence and risk factors of intestinal schistosomiasis in students at Bochesa Elementary School in the wetlands of Lake Ziway.

**Methods:**

A cross-sectional study of 384 students was conducted in May 2016. Intestinal schistosomiasis was examined using the Kato-Katz technique. For the association between the prevalence of intestinal schistosomiasis and categorical variables, the chi-square (χ^2^) test was used. A binary logistic regression was applied.

**Results:**

The overall prevalence of intestinal schistosomiasis was 25.52%. Males were more infected (17.45%) than women (8.07%), and the difference was significant (χ^2^ = 17.756; *P* <0.05). Lower grade (1–4) students were more infected (20.83%) than grade 5-8 students (4.69%), and the difference was significant (χ^2^ = 19.558; *P* <0.05). Age groups of 7-14-year-old students were more infected (24.74%) than >15-year-old students (0.78%), and the difference was significant (χ^2^ = 4.788; *P* <0.05).

**Conclusions:**

The result implied that the wetlands of Lake Ziway are conducive to schistosomiasis. Students should receive health education on the mode of schistosomiasis transmission and its prevention methods, and a buffer zone for the wetlands should be identified. Praziquantel treatment is crucial in reducing intestinal schistosomiasis in students.

## Highlights

• People living near wetlands rely on them to search for natural resources.
• Wetlands not only deliver resources but also spread diseases to the inhabitants.
• Intestinal schistosomiasis spreads through skin contact with contaminated wetland water.
• Wetland activities can cause schistosomiasis, particularly in school-aged children.
• The overall prevalence of intestinal schistosomiasis was 25.52%.

## Introduction

Around the world, early civilizations relied on wetland-based resources [1]. In developing countries such as Ethiopia, the growth of the human population necessitates closer interaction with wetlands as people seek natural resources for survival. In Ethiopia, wetland resources are considered public property, with unrestricted access granted to everyone. Wetlands provide economic and social value to surrounding people [2]. However, wetlands not only supply resources but; unfortunately, they also serve as sources of water-based diseases. Wetlands that provide resources to the surrounding inhabitants are also favorable habitats for trematode parasites in humans and their intermediate snail hosts [3]. Schistosomiasis is caused by Schistosoma species, which require intermediate snail hosts in wetlands for completion of their life cycle and to infect humans.

The spread of schistosomiasis between intermediate and definitive hosts relies on infective larval stages that swim freely and move through wetlands. In wetland areas, domestic activities, such as washing, swimming, fetching water, fishing, reed harvesting, engaging with moist soil, and crossing rivers with bare feet, expose individuals to schistosomiasis [4].

The prevalence of intestinal schistosomiasis varies among residents. The prevalence of intestinal schistosomiasis has been studied in several countries including Ethiopia [5,6]. Recent reports on surveys at the prevalence level in Sub-Saharan Africa indicated that Ethiopia has the highest schistosomiasis burden (24-76.3%) among students [7]. Therefore, this study aimed to assess the prevalence and risk factors of intestinal schistosomiasis in students living near the wetlands of Lake Ziway, Ethiopia.

## Methodology

### Study design, area, and period

In June 2016, a cross-sectional study was conducted among the students of Bochesa Elementary School, situated near the wetlands of Lake Ziway. Ziway Town lies southwest of Lake Ziway, approximately 167 km south of Addis Ababa, the capital city of Ethiopia. The school is proximal to the lake and located in the southern direction. The area is one of the hotspots for schistosomiasis, as students and their families depend on Lake Ziway and its outlet as a water source for everyday activities such as fishing, irrigation, swimming, and bathing in water containing infectious cercariae, which is considered a risk factor for intestinal schistosomiasis.

### Sample size and sampling technique

To assess the prevalence of intestinal schistosomiasis and related risk factors, the sample size (n) was calculated using a single population proportion formula [8].

$$n=\frac{\left(z^{2}p\left(1-p\right)\right)}{d^{2}}$$

As the prevalence of intestinal schistosomiasis in the study area was unknown to the students, a *P*-value of 50% was used. A 95% confidence interval (CI) (z) and 5% margin of error (d) were applied; therefore, a sample size of 384 students was included.

### Laboratory investigations

After obtaining oral consent from the school principal, each student was given a labeled plastic container, toilet tissue paper, and applicator sticks to bring her/his fresh stool sample.

Approximately 5 g of fresh stool samples were obtained from each student and placed in separate labeled clean plastic fecal containers. All fecal samples were processed within 30 minutes of collection using the modified Kato-Katz method. A small portion (2 g) of the sample was placed in a newspaper using an applicator stick, covered with a nylon screen, and pressed on top to sieve the stool through the nylon. A wooden spatula was used to collect the sieved stool sample and fill the hole of the Kato template placed on a microscopic slide. The template was carefully removed to keep the stool sample at the center of the slide. A cellophane strip pre-soaked in a glycerol-methylene blue solution was placed on the slide and left for 20 minutes for clarity. The examination was performed under 400 × magnification using a microscope. The total number of *Schistosoma mansoni* eggs observed was counted from the template and expressed as eggs per gram of stool (EPG) multiplied by 24.

### Questionnaire administration

A questionnaire was first prepared in English and then converted into Afan Oromo (the students’ local language) to obtain sociodemographic data and known potential risk factors for intestinal schistosomiasis.

### Statistical analysis

The prevalence of *S. mansoni* infections has been reported to vary. The chi-square (χ^2^) test was used to evaluate the association between categorical variables and prevalence.

For the identification of determinant factors, binary logistic regression was conducted, and the association between independent and dependent variables was described based on an odds ratio (OR) with a 95% CI. The crude OR was calculated using univariate regression analysis, and the adjusted OR was subsequently determined using multivariate logistic regression analysis. The results were considered statistically significant when the *P*-value was <0.05.

The intensity of infection was determined and interpreted by estimating the EPG of fecal samples based on established thresholds: 1-99 as light infection, 100-399 as moderate infection, and greater than 400 indicates heavy infection according to World Health Organization guidelines. To obtain the approximate number of EPG, the total number of intestinal Schistosoma eggs counted was multiplied by a factor of 24 [9].

## Results

Of the 384 students who participated in this study, 98 (25.52%, 95% CI: 21.10-29.68%) were found infected by intestinal schistosomiasis. The prevalences of intestinal schistosomiasis were statistically significant (χ^2^ = 17.556; *P* = 0.001, χ^2^ = 4.788; *P* = 0.018, χ^2^ = 19.558; *P* = 0.001, and χ^2^ = 5.229; *P* = 0.018) between sex, age, grade categories, and level of parents’ education, respectively (Table 1).

**Table 1:** The prevalence of intestinal schistosomiasis based on different variables in Bochesa Elementary school students at the Wetlands of Lake Ziway.

Variables	Frequency	Prevalence (%)	Chi-square value	*P*-value
Sex				
Male	67	17.45%	17.556	0.001
Female	31	8.02%		
Total	98	25.52%		
Age				
7-14	95	24.74%	19.558	0.001
>15	3	0.78%		
Total	98	25.52%		
Grade category				
1-4	80	20.83%	4.788	0.018
5-8	18	4.69%		
Total	98	25.52%		
Parents’ Education				
Illiterate	72	18.75%	5.229	0.018
Literate	26	6.77%		
Total	98	25.52%		

The EPG count ranged from 24-480, with arithmetic and geometric mean values of 156 EPG and 120.10 EPG, respectively. Intestinal schistosomiasis infection intensity was categorized as low, moderate, or heavy, with values of 68.37%, 27.55%, and 4.08%, respectively.

### Potential risk factors associated with intestinal schistosomiasis

Binary logistic regression was used to select the potential risk factors associated with intestinal schistosomiasis. Univariate binary logistic regression analysis indicated that sex (*P* = 0.001), grade category (*P* = 0.001), age (*P* = 0.039), level of parents education (*P* = 0.024), fishing habit (*P* = 0.001), the water source for bathing (*P* = 0.023), shoe-wearing habit (*P* = 0.001), the habit of playing with moist soil (*P* = 0.001), reed-harvesting habit (*P* = 0.001), place of defecation (*P* = 0.001), and toilet availability (*P* = 0.001) showed a significant association with intestinal schistosomiasis infection. In contrast, swimming habits and drinking water sources were not significantly associated with intestinal schistosomiasis (*P* >0.05).

Multivariate binary logistic regression revealed that students with literate parents had a 54.2% lower risk of intestinal schistosomiasis than those with illiterate parents (AOR: 0.458, 95% CI: 0.234-0.895). Students who had fishing habits were 3.963 times more likely to be infected by intestinal schistosomiasis than their counterparts (AOR: 3.963, 95% CI: 1.604-9.793). Students without a shoe-wearing habit were 2.786 times more likely to be infected with intestinal schistosomiasis than students who wore shoes (AOR: 2.786, 95% CI: 1.415-5.483). Students who frequently played with moist soil had a 3.042 times increased risk of intestinal schistosomiasis than students who did not engage in this activity (AOR: 3.042, 95% CI: 1.561-5.927). Similarly, students who had reed-harvesting habits were 3.002 times increased risk of intestinal schistosomiasis than students who did not have grass-cutting habits (AOR: 3.002, 95% CI: 1.283-7.026) (Table 2).

**Table 2:** Binary logistic regression analysis for factors potentially associated with intestinal schistosomiasis among students at Bochesa Elementary School.

Risk factors	Category	Positive (%)	Negative (%)	Adjusted odds ratio (95% confidence interval)
Sex	Male	67	125	1.353 (0.516-3.549)
	Female	31	161	
Grade category	1-4	80	162	1.576 (0.674-3.587)
	5-8	18	124	
Age	7-14	95	257	2.469 (0.537-11.341)
	>15	3	29	
Parents education	Illiterate	72	240	
	Literate	26	46	0.458^a^ (0.234-.0895)
Fishing habit	Yes	68	102	3.963 (0.981-7.062)
	No	30	184	
Water for bathing	Lake water	92	242	2.632 (0.720-6.274)
	Groundwater	6	44	
Shoe-wearing habit	Yes	31	184	
	No	67	102	2.786^a^ (1.415-5.483)
Playing with moist soil	Yes	36	35	3.042^a^ (1.561-5.927)
	No	62	251	
Reed harvesting habit	Yes	72	272	3.002^a^ (1.283-7.026)
	No	26	14	
Place of defecation	Latrine	9	151	
	Open area	74	150	1.673 (0.834-3.358)
Toilet availability	Yes	27	190	
	No	56	111	1.765 (0.904-3.445)

a Significant associations.

## Discussion

This study highlights the prevalence and intensity of intestinal schistosomiasis infection and its associated risk factors among students in the Lake Ziway wetlands. The prevalence of intestinal schistosomiasis observed in this study was 25.52% (95% CI: 21.10-29.68%). The results of this study are consistent with reports from within the country [10] and Uganda [11]. This finding was also higher than that reported in previous studies from different areas of the country [[12], [13], [14]] and abroad [15]. The result obtained was also lower than that of a baseline survey conducted on school children in Ethiopia [16]. This variation might be explained by differences in the sample size, study design, agroecological and climatic conditions, living conditions, and immune status of the study participants.

The prevalence of intestinal schistosomiasis was higher in men (17.45%) than in women (8.02%). In line with this finding, other reports within the country [17] and elsewhere in Africa have shown a high prevalence of intestinal schistosomiasis [5,11,18]. A possible reason for this result might primarily be the differences in daily activities and exposure patterns. The involvement of men in wetland field activities and water contact increased the time spent in contaminated water, leading to a higher likelihood of schistosomiasis infection.

The prevalence of intestinal schistosomiasis was also higher (24.74%) in the younger age group (7-14 years) than in their older counterparts (0.78%). Similar findings have been reported inside the country [13,14], and abroad [[19], [20], [21]]. Similarly, the prevalence of intestinal schistosomiasis in grades 1-4 was higher (20.83%) than in grades 5-8 (4.69%). Behavioral and immunological factors may explain the high prevalence of intestinal schistosomiasis in the lower grades. In lower age groups, due to a lack of awareness of the mode of transmission of the disease, as well as the frequent habit of recreational activities such as swimming and bathing in Lake Ziway wetland, where parasitic snails are present, they may be exposed to cercarial contamination. In addition, when students in lower grades spend a lot of time in contact with freshwater bodies for recreation, they are more likely to be infected. This finding is in agreement with other reports from Cote d'Ivoire [22,23].

The prevalence of intestinal schistosomiasis in students with illiterate parents was higher (18.75%) than in students with literate parents (6.77%). The higher infection rate observed in students with illiterate parents might be due to a lack of awareness, prevention, and health risks associated with frequent contact with cercaria-contaminated water. In line with this finding, research conducted in Pakistan indicated that schoolchildren of illiterate fathers were more likely to be infected with intestinal parasites than their counterparts [24]. Regarding the cause of schistosomiasis transmission, parental illiteracy leads to an absence of advice for their children. Therefore, the absence of awareness among illiterate parents and a lack of advice to their children may predispose students to intestinal schistosomiasis infection.

The intensity of infection observed in this study was low (68.37%), followed by moderate and heavy infection (27.55% and 4.08 %, respectively). This finding is consistent with the reports inside the country, which showed low infection (70.5%, 90.9%, and 71.1%), moderate (21.6%, 9.1% and 28.9%) and heavy (7.8%, 0%, and 0%) [13,16,25], respectively, and from Uganda, which showed low (62.7%), moderate (27.4%) and heavy infections (9.8%) [21].

This study showed that the likelihood of acquiring intestinal schistosomiasis with literate parents was 54.2% less than that among their counterparts. Similar findings were reported in Cote d'Ivoire [26], and Nigeria [27]. The reason for this might be that educated parents can understand how schistosomiasis is transmitted and thus can explain it to their children.

Students involved in fishing were more likely to be infected with intestinal schistosomiasis than students who did not have this habit. In this study, fishing activities held at the lake borders, the outlet of the lake, and irrigation canals also served as common defecation places for fishermen during working hours; therefore, transmission of intestinal schistosomiasis would be facilitated. Similar findings have been reported for other countries [28] and Sudan [29].

Students who did not wear shoes were more likely to be infected with intestinal schistosomiasis than those who wore shoes. This is because the mode of transmission of schistosomiasis is cervical skin penetration; barefootedness predisposes to the infection. This result is consistent with other findings from Ethiopia [28,30,31].

Students who frequently played with moist soil also had a higher risk of acquiring intestinal schistosomiasis than their counterparts. This might be because moist soils are found near water sources such as lakes and rivers, which harbor infected snails responsible for creating a conducive environment for intestinal schistosomiasis [32].

Students who had reed-harvesting habits were more likely to acquire intestinal schistosomiasis than those who did not. Because reed harvesting is a repetitive and routine income-generating task for households, it may lead to prolonged exposure to cercarial-contaminated water. This finding is consistent with that of another report from Ethiopia [33]. According to these authors, activities held in wetland areas are performed barefoot to minimize the risk of sinking their shoes. This might be attributed to the fact that reed-harvesting activities are a potential risk factor for students’ exposure to free-swimming cercariae and subsequent infections.

In conclusion, this study revealed that the wetlands of Lake Ziway are conducive to the transmission of intestinal schistosomiasis among students who come into contact with freshwater environments. Because the freshwater of the wetlands of Lake Ziway is hot and the environment is humid, coupled with moist soil, it is a suitable environment for the infective stages of intestinal schistosomiasis.

## Declarations of competing interest

The authors have no competing interests to declare.
